# Sensitivity analysis of counterweight double-row pile deformation to weak stratum parameters

**DOI:** 10.1038/s41598-023-47473-2

**Published:** 2023-11-23

**Authors:** Qiongyi Wang, Yungang Niu, Fenghai Ma, Shasha Lu

**Affiliations:** 1 School of Mechanics and Engineering, Liaoning Technical University, Fuxin, 123000 China; 2 College of Architecture and Engineering, Dalian University, Dalian, 116622 China; 3 School of Civil Engineering, Liaoning Technical University, Fuxin, 123000 China

**Keywords:** Civil engineering, Engineering

## Abstract

In order to investigate the sensitivity of weak soil parameters on the deformation of balanced double-row piles, a case study was conducted in a deep foundation pit project in Shenzhen City. A variety of analysis methods, including numerical simulation, field measurements, orthogonal experiments, and theoretical analysis, were employed to analyze the impact of three weak soil parameters on the deformation of balanced double-row piles. The research results showed that the deformation of the front and back rows of piles exhibited overturning deformation, gradually decreasing with depth and reaching the maximum at the pile top due to the constraint effect of the balance platform. The numerical simulation results of horizontal displacements for the front and rear piles were in good agreement with the field measurements, confirming the accuracy and reasonableness of the numerical analysis model and parameter selection. Through a series of orthogonal numerical simulation experiments, it was determined that the cohesive strength (*C*) of soft layers, such as rockfill and silt, is a key factor, the internal friction angle (*φ*) is an important influencing factor, and the elastic modulus (*E*) is a general influencing factor. Theoretical analysis was employed to establish the relationship curve between each parameter and the maximum pile deformation, as well as the sensitivity factors, further verifying the impact of these weak soil parameters. The research findings presented in this paper can provide valuable guidance for geotechnical engineers when selecting geological parameters for similar deep excavation projects.

## Introduction

With the rapid development of coastal urbanization, the utilization of land reclamation and underground space has effectively addressed various urban land constraints. However, it has also presented numerous challenges in underground engineering, mainly characterized by complex geological conditions and high demands for controlling surrounding environmental deformations^1–6^. Therefore, it is necessary to conduct research on the impact of weak soil parameters on the deformation of supporting structures in reclaimed areas.

In recent years, as the number of deep excavation projects in weak soil areas has increased, significant progress has been made in this field of study. Xiao et al.^7^ based on a case study in a specific soft soil area, employed parameter sensitivity analysis to identify critical geological parameters. They proposed a dynamic construction inverse analysis method using parameter sensitivity analysis and BP (Backpropagation) neural networks. Qi et al.^8^ took the Shenzhen International Convention and Exhibition Center Project (Phase I) super-large deep foundation pit engineering design as the research object. In order to solve the challenges of tight construction schedule and high deformation control requirements in the reclamation area, a combined method of counterweight double-row piles and large-span supports was employed. A calculation model for the support stiffness of this structure was established, and numerical simulations were conducted to investigate the influence of large-span support spacing on the deformation and internal forces of the supporting structure. Lan et al.^9^ investigated the effects of dimensional parameters such as pile diameter and length, as well as engineering parameters such as pile spacing and row spacing, on the stress and deformation of double-row pile support structures. They also studied the influence of soil arching effect on the stress and deformation of these structures based on a calculation model considering pile-soil interaction. Li et al.^10^ effectively combined the treatment of soft soil foundation with the excavation and support of deep foundation pits, proposing a new method for excavating deep foundation pits in soft soil—the Vacuum Curtain Waterproofing and Atmospheric Pressure Support Deep Foundation Pit Excavation method. This method has provided new design concepts and directions for the development of deep foundation pit engineering technology. Based on the enclosed area of the foundation pit project for the Guangxi Nanning Rail Transit Line 4, Ou et al.^11^ used the finite element software ABAQUS to establish a numerical model of the deep foundation pit double-row pile support structure. They combined it with on-site measurement data to analyze the displacement and deformation characteristics of the double-row pile support structure. By changing the spacing between the double-row piles, the pile diameter, the pile stiffness, and the beam height, they analyzed the impact of design parameter changes on the support effect. Yan et al.^12^ conducted a three-dimensional elastoplastic numerical simulation of the excavation process for the maximum excavation depth of the double-row pile support in the underground water treatment plant foundation pit project in Wuhan City. By comparing the field measurement results, design software calculation results, and numerical calculation results, they validated the rationality of the numerical model. They analyzed the effects of pile-soil reinforcement depth, elastic modulus, cohesion, and internal friction angle on the bearing deformation characteristics of the double-row pile support system. Dong et al.^13^ derived and studied the theoretical calculations of the counterweight double-row pile support structure. They applied the derived calculation method to Midas GTS NX simulation calculations. Taking a deep foundation pit project in Shenzhen City as an example, they verified and analyzed the simulation results and field monitoring results. Based on this, they further explored the influence of deformation parameters such as row spacing, diameter of back row piles, and top load on the pile body. Meng et al.^14^, based on a certain deep foundation pit project in a soft soil area, established a three-dimensional foundation pit numerical model. They analyzed the influence of the dimensions, specifically the width and depth of the foundation pit model, on the deformation during excavation. Their findings suggest that when the width of the foundation pit model is greater than 7 times the excavation depth and the depth exceeds 6 times the excavation depth, the influence of the size effect on the model can be essentially disregarded. Ren et al.^15^ conducted on-site monitoring of the foundation pit's groundwater level, pore water pressure, and dewatering well flow rates to analyze the temporal and spatial variations in dewatering. They further investigated the deformation and seepage characteristics induced by excavation-induced dewatering by analyzing the measured results of soil settlement and horizontal deformation of the retaining structure. Zhang et al.^16^ based on the Euler–Bernoulli double-layer subgrade beam theory, considered the interaction between piles and pile-soil, and obtained the flexural differential equations of the double-row pile support structure in layered subgrade. They solved the equations using the power series method and obtained the calculation formulas for the horizontal displacement, rotation angle, shear force, and bending moment of the pile body at any depth based on the continuity conditions of internal forces and displacements at the interface and the boundary conditions at the pile top and pile bottom. Finally, through an engineering case analysis, they compared and analyzed the calculation results of their method with the results of the case study, validating the correctness of their method. Yi et al.^17^ studied the influence of retained soil in the pit on the double-row pile support structure. They used the finite element numerical analysis method to parametrically analyze the dimensions of the retained soil, reinforcement effects, and elastic modulus, and conducted an in-depth study on the displacement, bending moment, and passive zone soil pressure changes of the double-row piles. Fan et al.^18^ employed finite element analysis considering the small strain stiffness characteristics of soil in multiple case statistics to investigate the influence of internal support pit excavation on the deformation characteristics of the underlying subway tunnels. They defined influence area parameters based on deformation contour analysis, combined with deformation control standards of 20 mm, 10 mm, and 5 mm for the subway tunnels, and established the corresponding impact zone ranges. They also simplified the description of the impact zone range based on its characteristics. Niu et al.^19^ conducted a study to investigate the influence characteristics of reverse faults on the deformation of pile foundations in deep excavation projects. They used various research methods such as numerical simulation, field monitoring, and orthogonal experiments, taking a deep excavation project in Shenzhen as an example. They analyzed the effects of reverse faults on the deformation of retaining piles, finding a positive correlation between pile deformation and fault slip distance as well as fault dip angle. Furthermore, they analyzed the sensitivity of fault slip distance, fault dip angle, and fault position to the maximum deformation of the piles. They determined that fault dip angle had the highest sensitivity, followed by fault slip distance, while fault position had the lowest sensitivity.

Through an analysis of the literature mentioned above, it becomes evident that there is relatively limited research regarding the influence of weak soil parameters on the deformation of supporting structures in reclaimed areas. Therefore, in this study, based on a deep pit engineering project in Shenzhen, orthogonal numerical simulation experiments were conducted using MIDAS GTS NX finite element software to analyze the sensitivity of three parameters of weak soil to the deformation of cantilevered double-row pile piles. The research findings have certain theoretical value and practical significance for engineering applications.

## Project introduction

**Figure 1:**
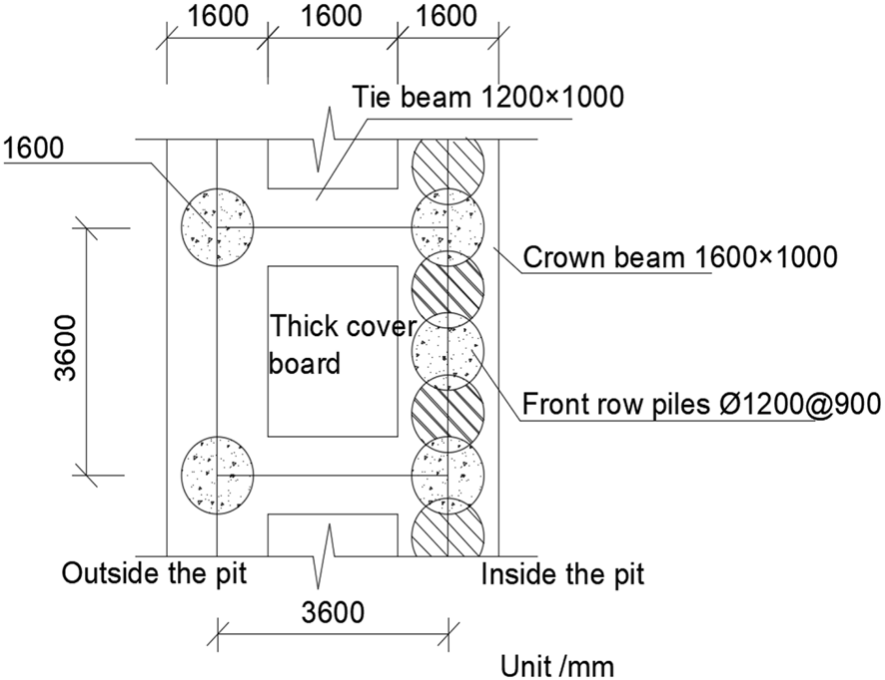
Plan view schematic diagram of double-row piles.

This project is located in the Shenzhen Bay reclamation area of Nanshan District, Shenzhen City. The subsurface strata at the site consist of fill stone, silt, silty clay, gravelly clay, and completely weathered granite, arranged from top to bottom. The pit design employs a cantilevered double-row pile support structure. The front row piles are *ϕ*1.2 m@1.8 m interlocking piles arranged in an alternating pattern, while the back row piles are *ϕ*1.2 m@3.6 m single piles. The plan view schematic diagram of double-row piles is shown in Fig. 1. The embedment depth of the front row single piles is 13.0 m, and the embedment depth of the back row single piles is 15.0 m. The spacing between piles is 3.6 m. The cross-sectional dimensions of the cap beams are 1.6 m × 1.0 m, and the dimensions of the connecting beams are 1.2 m × 1.0 m. The *L*-shaped counterweight platform consists of a horizontal plate approximately 4.8 m wide and 0.5 m thick, along with retaining plates that are 2.3 m high and 0.3 m thick on the upper part of the horizontal plate. The *L*-shaped counterweight platform is used for backfilling soil and unloading pressure. The section dimensions and physical and mechanical performance parameters of the supporting structure are shown in Table 1.

**Table 1:** The cross-sectional dimensions and physical–mechanical performance parameters of the support structure.

Name	Material	Section dimensions/mm	Elastic modulus/GPa	Poisson’s ratio
Front (back) row of piles	C30	***ϕ*** 1200	30	0.2
Crown beam	C30	1600 × 1000	30	0.2
Waist beam	C30	1200 × 1000	30	0.2
L-shaped weighing table horizontal plate	C30	4800 × 500	30	0.2
L-shaped counterweight retaining plate	C30	2300 × 300	30	0.2

**Figure 2:**
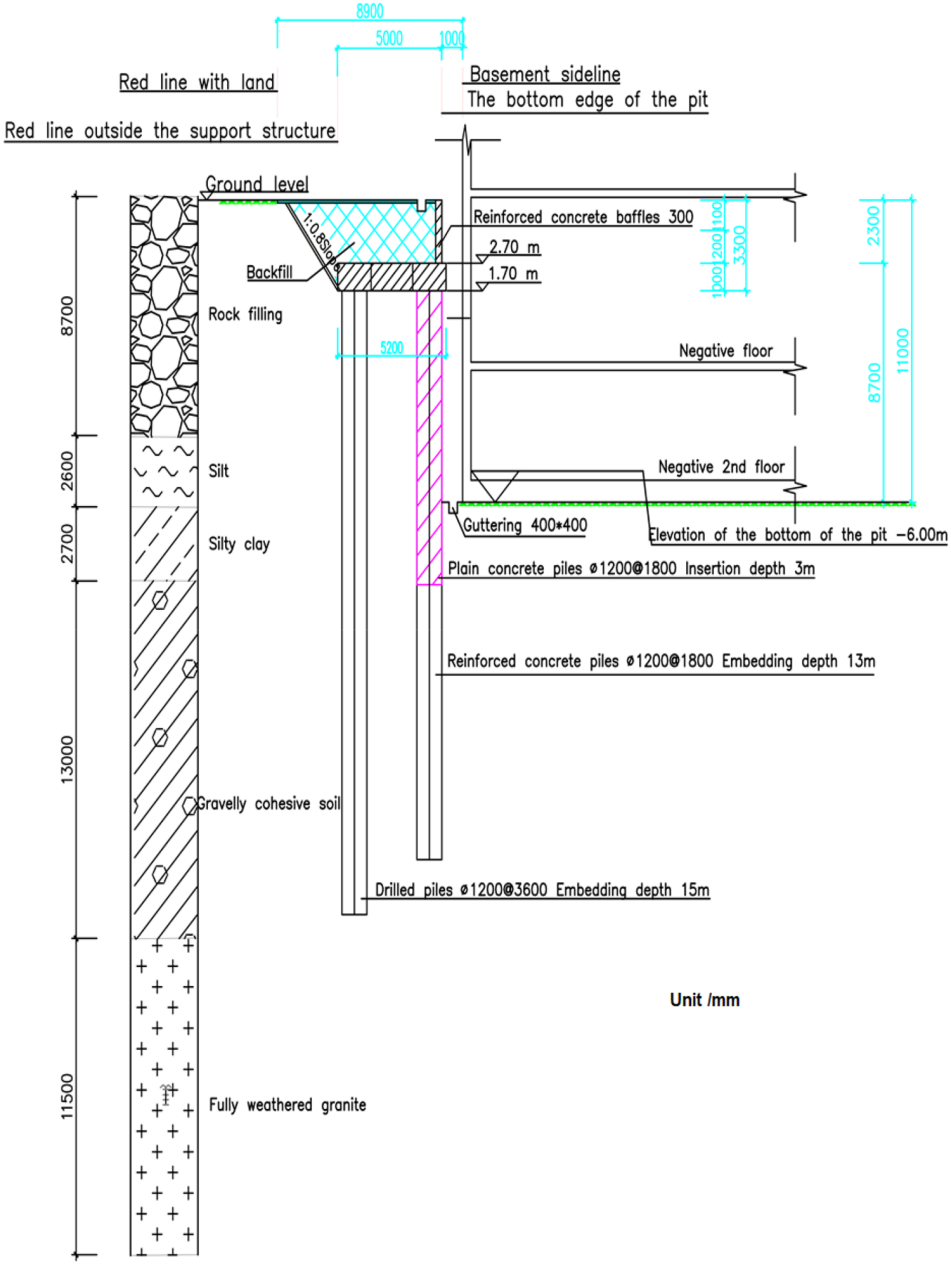
Design diagram of double-row pile.

**Figure 3:**
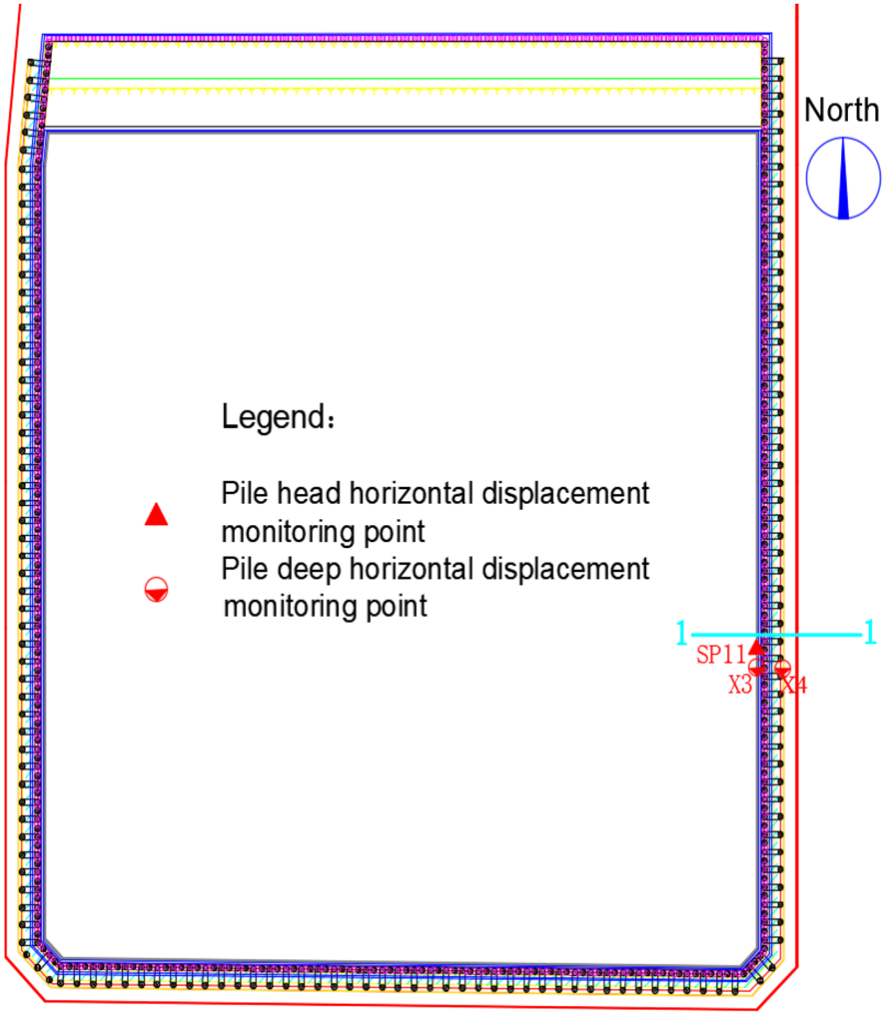
Layout of on-site measurement points.

This paper selected the profile with the worst geological conditions for analysis, which is the eastern profile 1–1 of the foundation pit, as shown in Fig. 2. The actual plan excavation dimensions of the foundation pit are 250 m × 170 m, with a maximum excavation depth of 11.0 m. The foundation pit is excavated in three layers, with the first layer excavated to a depth of 2.7 m, the second layer to a depth of 8.6 m, and the third layer to the bottom of the pit. According to the Pit Engineering Handbook and relevant regulations for pit engineering in the Shenzhen area^20,21^, monitoring points for pile deformation in the cantilevered double-row pile configuration were set up at the site. Since this study focuses on the eastern profile 1–1 with the worst geological conditions, the layout diagram for on-site monitoring points only displays the monitoring points for this specific profile. The layout of on-site monitoring points is shown in Fig. 3.

## Three-dimensional numerical simulation analysis

### Establishment of finite element model and selection of calculation parameters

**Figure 4:**
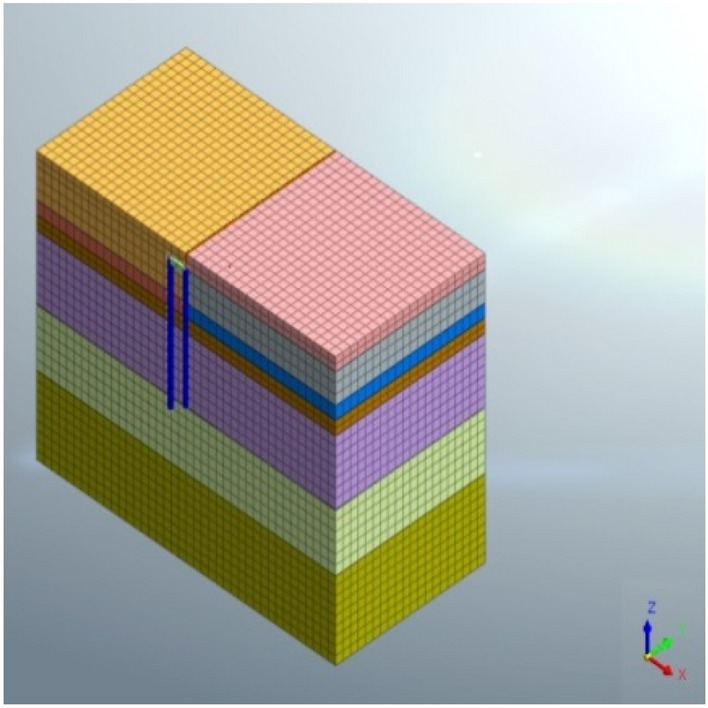
Three dimensional finite element model.

**Figure 5:**
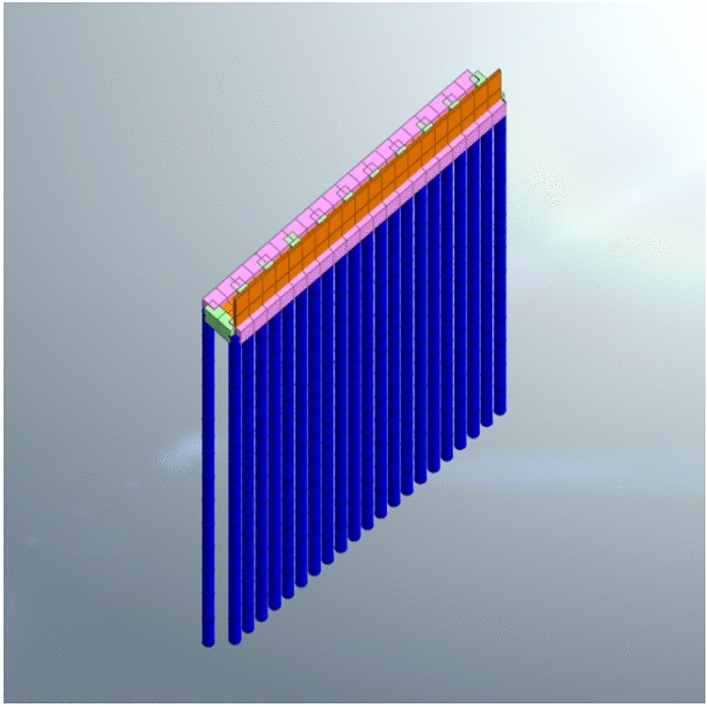
Support structure model.

A three-dimensional numerical simulation analysis model was established using the finite element software MIDAS GTS NX to analyze the excavation stages of the foundation pit. The dimensions of the soil model were 72 m horizontally along the 1–1 profile, 36 m vertically perpendicular to the 1–1 direction, and 54 m vertically, resulting in a model size of 72 m × 36 m × 54 m.The support piles were represented using one-dimensional implanted beam elements, while the crown beam and connecting beam were modeled using one-dimensional elastic beam elements. The counterweight plate and retaining plate were simulated using plane plate elements. The soil was modeled using a modified Mohr–Coulomb constitutive model, and the parameter values for each soil layer are shown in Table 2, with an elastic modulus of C30 concrete taken as 3 × 10^4^ MPa, a Poisson's ratio of 0.20, and a density of 25kN/m^3^. The boundary conditions of the calculation model were set as: vertical displacement constraints at the bottom and horizontal constraints around the model, limiting horizontal and vertical displacement. The entire model was divided into a regular hexahedral mesh with a total of 26,366 mesh units and 27,616 node elements. The three-dimensional finite element analysis model and support structure model are shown in Figs. 4 and 5.

**Table 2:** Parameter values of each soil layer.

Stratigraphic(Genetic)	Natural weight (kN/m^3^)	Elasticity modulus (MPa)	Cohesion (kPa)	Internal friction angle (◦)	Permeability coefficient (m/d)
1–1 Rockfill (Q_4_^mL^)	20.0	106	6	32	3.0
2–1 Silt (Q^m^)	16.5	10	10	6	0.005
3–1 Silty clay(Q_4_^al+pl^)	18.5	45	20	16	0.05
4–1 Gravel clay (Q^el^)	18.5	54	21	23	0.1
5–1 Completely weathered granite (γK_1_)	19.0	150	23	28	0.2

### Numerical simulation results

**Figure 6:**
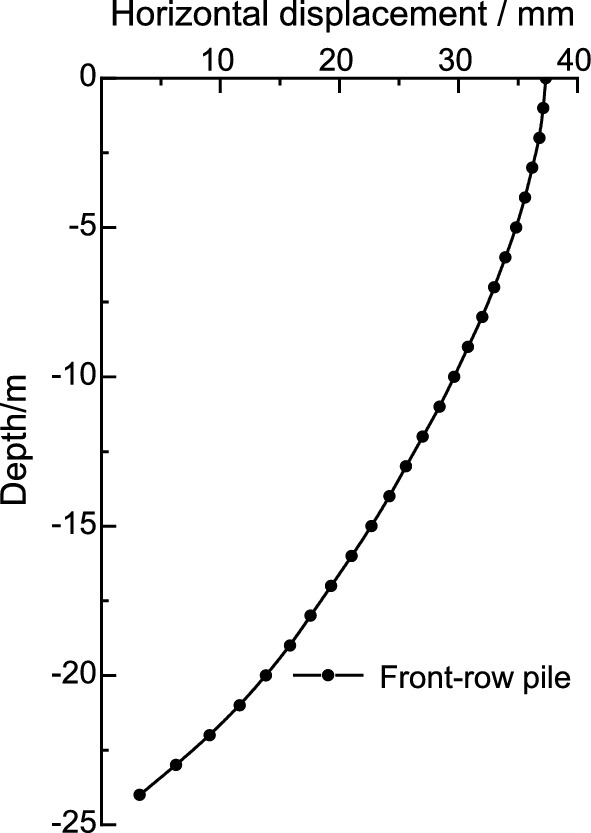
Horizontal displacement of deep layer of the front-row pile.

**Figure 7:**
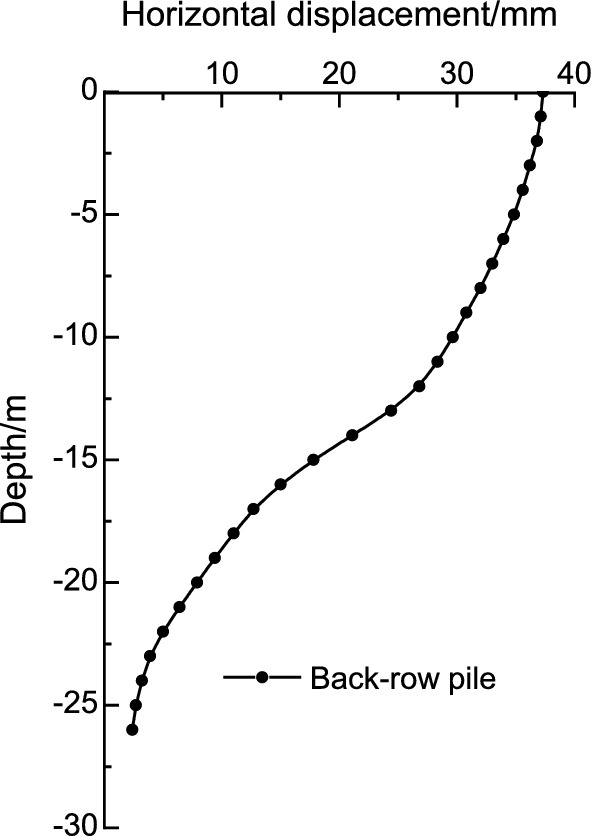
Horizontal displacement of deep layer of the back-row pile.

After excavation of the foundation pit to the bottom, the deep horizontal displacement variation curves of the pile bodies at X3 and X4 of the front and rear rows of piles were extracted, as shown in Figs. 6 and 7. Both the top and bottom of the piles have a certain amount of horizontal displacement, and overall, the deformation of the front and rear piles is of the tilting type. Due to the constraint of the counterweight platform, the deep horizontal displacement deformation pattern of the front and rear piles is basically identical, gradually decreasing with increasing depth. The maximum deep horizontal displacement of the front and rear piles occurs at the pile top, and the horizontal displacement values at the pile bottom and top are essentially equal.

## Comparison and analysis of numerical simulation and field measurement data

**Figure 8:**
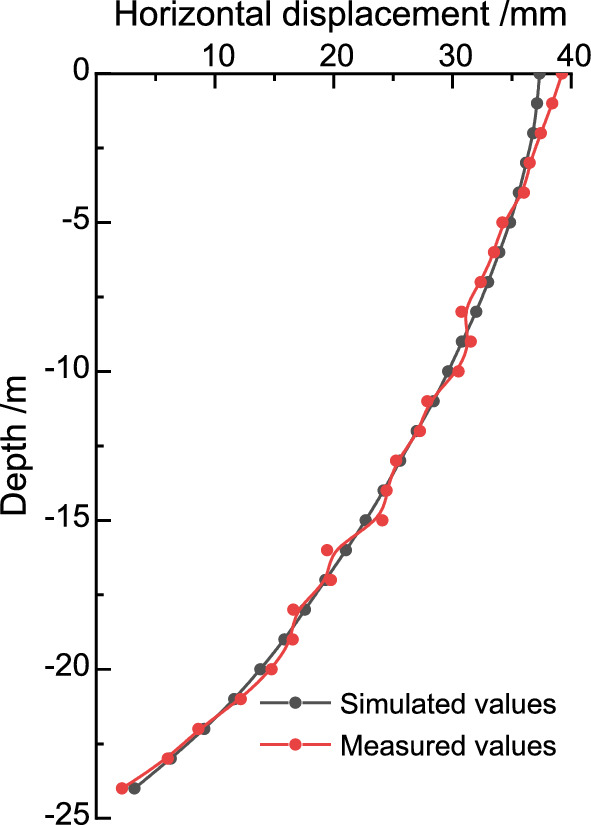
Comparison of deep horizontal displacement of front-pile in the third stage.

**Figure 9:**
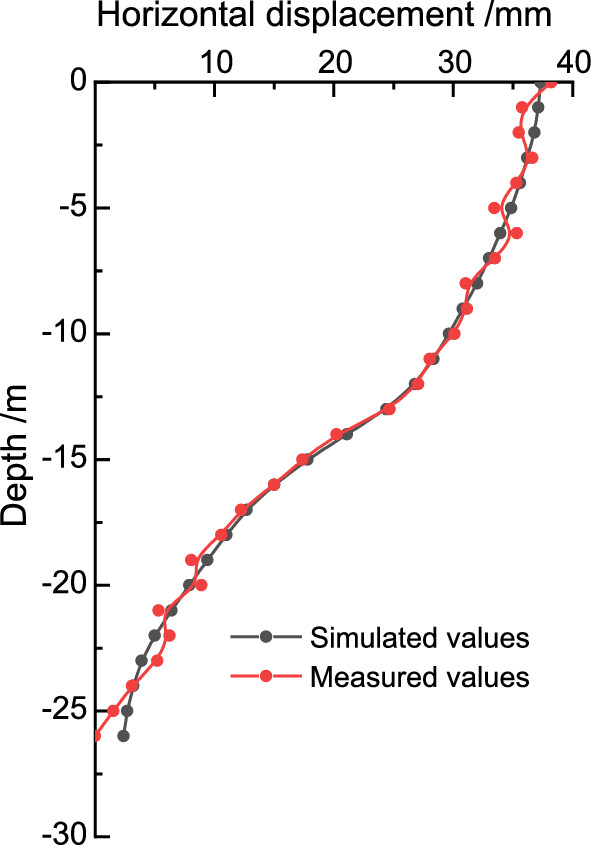
Comparison of deep horizontal displacement of back-pile in the third stage.

The deep horizontal displacement of the front row pile at the on-site measurement point X3, and the deep horizontal displacement of the back row pile at the on-site measurement point X4 are shown in Fig. 3. The results of the comparison between the finite element calculation values and the field measurement data after excavation are shown in Figs. 8 and 9. The deep horizontal displacement deformation law of the front and rear pile bodies obtained by finite element analysis is basically consistent with the field measurement values. The horizontal displacement calculation results at the pile bottoms of both the front and back rows exceed the measured data, while the horizontal displacement calculation results at the pile tops are smaller than the measured data. As the excavation depth of the foundation pit increases, the horizontal displacement of the front and back row piles at deeper levels also increases. It should be noted that the measured results of horizontal displacement at the bottom and top of the pile may be affected by factors such as the excavation of layered zones, surrounding loading, and delayed reinforcement of support, which were not taken into account. This may lead to certain deviations in the results. Overall, the numerical results of both parameters generally agree, indicating that finite element analysis can to some extent reflect the actual situation.

## Sensitivity analysis

### Orthogonal experimental analysis

In this paper, the authors employ an orthogonal experimental design to examine the impacts of cohesion (*C*), internal friction angle (*φ*), and elastic modulus (*E*) on numerical simulations involving two weak layers: filling stones and silt. A three-factor, five-level orthogonal numerical simulation experiment is conducted, comprising a total of 25 experiment groups. Aside from the previously mentioned parameters associated with weak soil conditions, the models and calculation parameters remain consistent for each test group. Specific parameter values for each factor are detailed in Tables 3, 4 and 5.

**Table 3:** Parameters of cohesiveness of soil.

Stratigraphic(Genetic)	Cohesion*/*kPa				
	A1	A2	A3	A4	A5
1–1 Rockfill (Q_4_^mL^)	3.0	4.0	5.0	6.0	7.0
2–1 Silt (Q^m^)	7.0	8.0	9.0	10.0	11.0
3–1 Silty clay(Q_4_^al+pl^)	20.0	20.0	20.0	20.0	20.0
4–1 Gravel clay (Q^el^)	21.0	21.0	21.0	21.0	21.0
5–1 Completely weathered granite (γK_1_)	23.0	23.0	23.0	23.0	23.0
1–1 Rockfill (Q_4_^mL^)	25.0	25.0	25.0	25.0	25.0

**Table 4:** Parameters of internal friction Angle of soil.

Stratigraphic(Genetic)	Internal friction angle/°				
	B1	B2	B3	B4	B5
1–1 Rockfill (Q_4_^mL^)	28.0	29.0	30.0	31.0	32.0
2–1 Silt (Q^m^)	2.0	3.0	4.0	5.0	6.0
3–1 Silty clay(Q_4_^al+pl^)	16.0	16.0	16.0	16.0	16.0
4–1 Gravel clay (Q^el^)	23.0	23.0	23.0	23.0	23.0
5–1 Completely weathered granite(γK_1_)	28.0	28.0	28.0	28.0	28.0
1–1 Rockfill (Q_4_^mL^)	32.0	32.0	32.0	32.0	32.0

**Table 5:** Parameters of elastic modulus of soil.

Stratigraphic(Genetic)	Elasticity modulus*/*MPa				
	C1	C2	C3	C4	C5
1–1 Rockfill (Q_4_^mL^)	103	104	105	106	107
2–1 Silt (Q^m^)	7	8	9	10	11
3–1 Silty clay(Q_4_^al+pl^)	45	45	45	45	45
4–1 Gravel clay (Q^el^)	54	54	54	54	54
5–1 Completely weathered granite (γK_1_)	150	150	150	150	150
1–1 Rockfill (Q_4_^mL^)	240	240	240	240	240

Based on the data provided above, the orthogonal experimental factors and levels are shown in Table 6. A total of 25 numerical simulation scenarios can be derived from the orthogonal experimental design scheme and parameter values. These simulations were conducted using MIDAS GTS NX for numerical analysis. The range is denoted as "*R*" and represents the difference between the maximum and minimum values of the sum of results caused by variations in the order of parameters such as cohesion (*C*), internal friction angle (*φ*), elastic modulus (*E*), and others. Specifically, it refers to the difference between the maximum and minimum values of the sum of the maximum horizontal displacements of the front and rear piles resulting from different orders, including A1 to A5, B1 to B5, and C1 to C5, as shown in Table 7.

**Table 6:** Orthogonal test factors and levels.

Factors levels	Cohesion*/*kPa	Internal friction angle/(°)	Elasticity modulus*/*MPa
1	A1	B1	C1
2	A2	B2	C2
3	A3	B3	C3
4	A4	B4	C4
5	A5	B5	C5

**Table 7:** Orthogonal experimental design scheme and simulation results.

Trial number	Factor 1		Factor 2		Factor 3		Maximum horizontal displacement of front-row piles/mm	Maximum horizontal displacement of back-row piles/mm
1	A1		B1		C1		42.13	42.10
2	A1		B2		C2		40.39	40.35
3	A1		B3		C3		38.8	38.77
4	A1		B4		C4		37.37	37.33
5	A1		B5		C5		36.05	36.02
6	A2		B1		C2		40.20	40.16
7	A2		B2		C3		38.6	38.57
8	A2		B3		C4		37.18	37.14
9	A2		B4		C5		35.87	35.83
10	A2		B5		C1		37.76	37.73
11	A3		B1		C3		38.37	38.34
12	A3		B2		C4		36.94	36.91
13	A3		B3		C5		35.63	35.60
14	A3		B4		C1		37.5	37.47
15	A3		B5		C2		36.09	36.06
16	A4		B1		C4		36.62	36.62
17	A4		B2		C5		35.34	35.31
18	A4		B3		C1		37.21	37.18
19	A4		B4		C2		35.8	35.77
20	A4		B5		C3		34.51	34.48
21	A5		B1		C5		35.03	35
22	A5		B2		C1		36.88	36.85
23	A5		B3		C2		35.48	35.45
24	A5		B4		C3		34.21	34.18
25	A5		B5		C4		33.06	33.03
	Front	Back	Front	Back	Front	Back		
Range *R*	20.08	20.06	17.88	17.90	13.56	13.57		
Relative range(%)	10.88	10.87	9.68	9.70	7.29	7.36		

### Orthogonal experimental results

**Figure 10:**
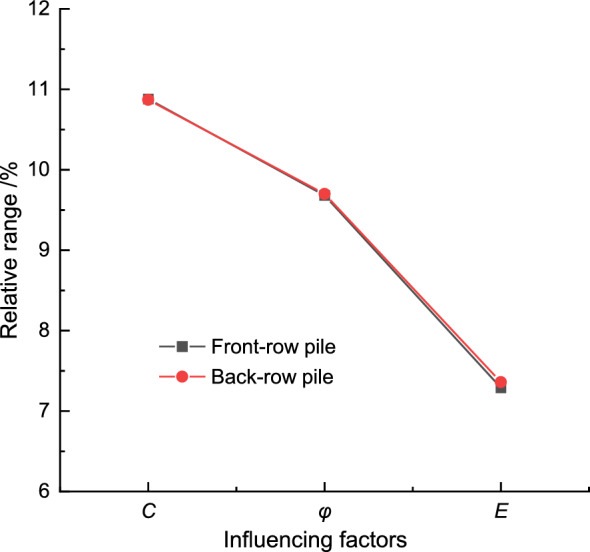
Comparison of relative range of each influencing factor of front-pile.

This paper employs the relative range of maximum horizontal pile body displacement from orthogonal experimental results to investigate the sensitivity of balanced double-row pile deformation to weak stratum parameters. The results demonstrate that the larger the relative range, the greater the impact of the corresponding factors on the target parameters, and vice versa. Analyzing the calculation results and the relative range value curve of influencing factors for the front and back row piles in Fig. 10, it becomes apparent that in the front row piles, the cohesive force (*C*) factor of the soil exhibits the largest relative range value at 10.88%, making it the key factor. The internal friction angle (*φ*) factor of the soil has an important influence with a relative range value of 9.68%, while the elastic modulus (*E*) factor of the soil plays a general influencing role with a relative range value of 7.29%. Similarly, the analysis of influencing factors for the back row piles yields consistent results with those of the front row piles.

### Theoretical analysis method

Assuming a system, the system variable is *Q*, which is determined by *m* main factor parameters *β*_*1*_, *β*_*2*_, *β*_*3*_, … , *β*_*m*_. The relationship between the variable *Q* and the parameters can be expressed as *Q* = *f* (*β*_*1*_, *β*_*2*_, *β*_*3*_, …, *β*_*m*_). Assuming that the parameters under certain conditions are set as the reference parameters, the system reference variable Q^*^ can be obtained as Q^*^ = *f* (*β*_1_^*^, *β*_2_^*^, *β*_3_^*^, …, *β*_m_^*^). When multiple factor parameters are adjusted, the trend and degree of deviation of the system variable *Q* from the reference variable Q^*^ indicate the sensitivity of parameter influence^22–24^.

In order to compare the sensitivity of multiple parameters, sensitivity functions and sensitivity factors in dimensionless form are defined. The relative error of the system variable *Q* and the relative error of parameter *β*_*k*_ are defined as the sensitivity function *S*_*k*_(*β*_*k*_). When △*β*_*k*_/△Q is small, *S*_*k*_(*β*_*k*_) can be approximately expressed as:

## Theoretical analysis results

The baseline model and parameters established in this section are consistent with those in Sect. 3. The experimental design involved changing one parameter while keeping the other two constant, based on the values from Tables 3, 4 and 5. Due to space constraints, this section only conducts sensitivity analysis on cohesion (*C*), internal friction angle (*φ*), and elastic modulus (*E*) for the silty clay strata. The initial reference values for parameters *C*, *φ*, and *E* are based on the geotechnical test report. The specific numerical values can be found in Tables 2, 3, 4 and 5. The sensitivity factor is the first-order derivative of the maximum pile deformation with respect to the parameter values mentioned above.

1. Sensitivity analysis of cohesive *C.*

**Figure 11:**
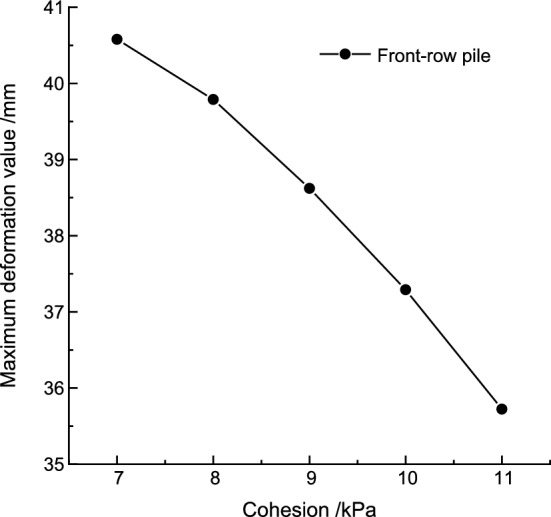
Relationship between pile maximum deformation and cohesion.

Keeping the internal friction angle and elastic modulus constant, the maximum deformation of the pile body under different cohesion values is shown in Table 8. The relationship between the maximum pile deformation and cohesion is illustrated in Fig. 11. The maximum pile body deformation decreases as the cohesion increases. The polynomial function curve that fits the relationship between the maximum pile body deformation (*μ*) and the cohesion is:

**Table 8:** The maximum deformation of front row piles under different cohesion.

Cohesive *C*/kPa	7	8	9	10	11
Maximum deformation /mm	40.58	39.79	38.62	37.29	35.72

(2) Sensitivity analysis of internal friction angle *φ.*

**Figure 12:**
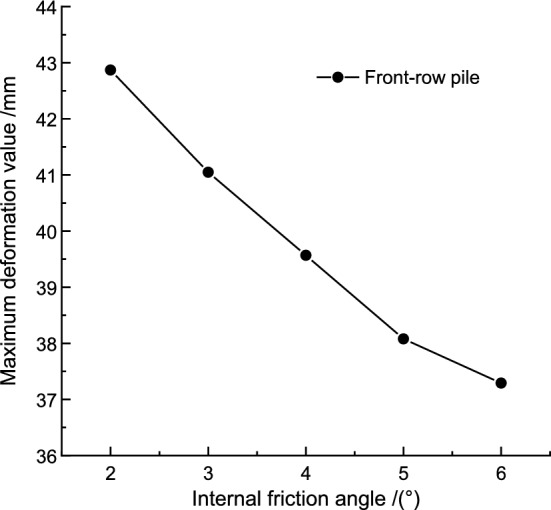
Relationship between pile maximum deformation and internal friction angle.

Keeping the cohesion and elastic modulus constant, the maximum deformation of the pile body under different internal friction angles is shown in Table 9. The relationship between the maximum pile deformation and internal friction angle is illustrated in Fig. 12. The maximum pile body deformation decreases as the internal friction angle increases. The polynomial function curve that fits the relationship between the maximum pile body deformation and the internal friction angle is:

**Table 9:** The maximum deformation of front row piles under different internal friction angles.

Internal friction angle *φ*/(◦)	2	3	4	5	6
Maximum deformation /mm	42.87	41.05	39.57	38.08	37.29

(3) Sensitivity Analysis of Elastic Modulus *E.*

**Figure 13:**
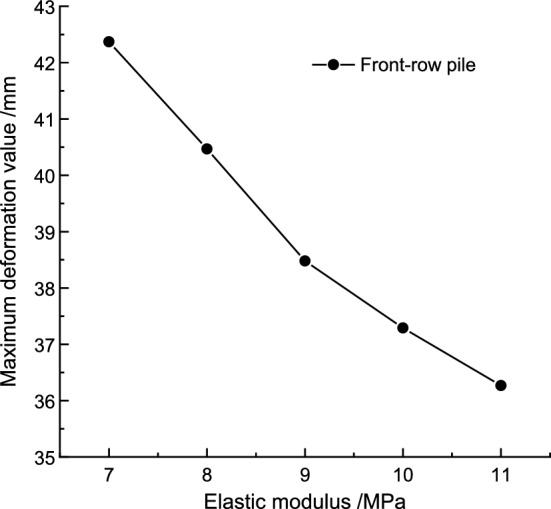
Relationship between pile maximum deformation and elastic modulus.

Keeping the cohesion and internal friction angle constant, the maximum deformation of the pile body under different elastic modulus values is shown in Table 10. The relationship between the maximum pile deformation and elastic modulus is illustrated in Fig. 13. The maximum pile body deformation decreases as the elastic modulus increases. The polynomial function curve that fits the relationship between the maximum pile body deformation and the elastic modulus is:

**Table 10:** The maximum deformation of front row piles under different elastic modulus.

Elastic modulus *E*/MPa	7	8	9	10	11
Maximum deformation /mm	42.37	40.47	38.48	37.29	36.26

In summary, Table 11 displays the sensitivity factors of various parameters to the maximum deformation of the double-row pile body. The cohesion (*C*) value has a sensitivity factor of 0.393, designating it as the key factor. The internal friction angle (*φ*) value has a sensitivity factor of 0.369, making it an important factor. Additionally, the elastic modulus (*E*) value has a sensitivity factor of 0.312, categorizing it as a general factor. All three parameters exert certain effects on the deformation of the double-row pile body. Therefore, when designing the support structure values, it is essential to comprehensively consider the influence of each parameter.

**Table 11:** Summary table of sensitive factors.

Indicator	Maximum deformation sensitivity factor of pile shaft
Cohesion (*C*)	0.393
Internal friction angle (*φ*)	0.369
Elasticity modulus (*E*)	0.312

## Conclusion and outlook

Taking a deep foundation pit engineering project in Shenzhen Bay reclamation area, Nanshan District, Shenzhen City as the research subject, a comprehensive analysis was conducted using methods such as numerical simulation, field measurements, orthogonal experiments, and theoretical analysis to perform sensitivity analysis on three weak soil parameters affecting the deformation of the cantilevered double-row piles. The main conclusions are as follows:

1. Both the top and bottom of the front and back row piles exhibit certain horizontal displacements, with the overall deformation being characterized as cantilever-type deformation. Due to the restraining effect of the counterweight platform, the deep-seated horizontal displacement of the front and back row piles follows a similar pattern, gradually decreasing with depth. The maximum deep-seated horizontal displacement of both the front and back row piles occurs at the pile top, with the horizontal displacement values at the pile bottom and pile top being roughly equal.
2. A three-dimensional numerical simulation analysis model for pit excavation was established using the finite element analysis software MIDAS GTS NX. A comparison between numerical simulation results and on-site measurements indicates that the numerical analysis results are in good agreement with the actual field measurements, validating the rationality and accuracy of the model and parameter selection in this study.
3. Sensitivity analysis was conducted using the orthogonal experimental method, and through a three-factor, five-level orthogonal numerical simulation experiment, a total of 25 groups of maximum pile deformation values for the front row piles were obtained. By calculating and utilizing the relative range, the sensitivity of various weak soil parameters on the horizontal deformation of the front row piles during pit excavation was studied. The results show that cohesion (*C*) in weak soil layers such as fill stone and silt is a critical factor, internal friction angle (*φ*) is an important influencing factor, and elastic modulus (*E*) is a generally significant factor.
4. Sensitivity analysis was performed using the theoretical analysis method. By controlling variables, relationship curves between various weak soil parameters and maximum pile deformation were obtained. These curves were then fitted, and sensitivity factors were calculated, further validating the impact of these weak soil parameters.

This paper only considers the influence of soft soil parameters on the deformation of cantilevered double-row piles, while there are many other factors affecting the deformation of deep foundation pit engineering in weak soil conditions that require further investigation. These factors include pile spacing, the depth and width of *L*-shaped counterweight platforms, as well as the spatiotemporal effects of soil parameters.

## Data Availability

The datasets used and/or analyzed during the current study are available from the corresponding author on reasonable request. The materials used in this study are commercially available or can be obtained from the corresponding author upon request. In addition, all relevant codes and protocols used in the study are available on GitHub and can be freely accessed and used for non-commercial purposes. The data that support the findings of this study are available from Shenzhen Dasheng Surveying Technology Co. but restrictions apply to the availability of these data, which were used under license for the current study, and so are not publicly available. Data are however available from the authors upon reasonable request and with permission of Shenzhen Dasheng Surveying Technology Co.
